# Commentary: GETgene-AI: a framework for prioritizing actionable cancer drug targets

**DOI:** 10.3389/fsysb.2026.1793501

**Published:** 2026-03-30

**Authors:** Ling Yin

**Affiliations:** 1 College of Life Science, Weifang Institute of Technology, Weifang, Shandong, China; 2 Weil Cornell Medicine, Cornell University, New York, NY, United States

**Keywords:** artificial intelligence, GETgene-AI, large language models (LLMs), precision oncology, target prioritization

## Introduction: the challenge of target prioritization in precision oncology

In the rapidly evolving field of precision oncology, systematically identifying and prioritizing actionable drug targets remains a critical and persistent challenge. The complexity and heterogeneity of cancers, particularly aggressive malignancies such as pancreatic ductal adenocarcinoma (PDAC), demand innovative computational approaches that can integrate multi-dimensional data and extract biologically meaningful insights beyond conventional analytical paradigms. A recent study by Gu and Chen introduces GETgene-AI, a novel and comprehensive computational framework designed to transcend traditional limitations through systematic integration of multi-modal data and the strategic application of artificial intelligence (Gu and Chen, 2025).

The methodological limitations of traditional target discovery approaches are well-documented. Many established methods, such as those based on fold-change differential expression analysis, are known to identify statistically significant changes that may lack biological or therapeutic relevance. As highlighted by McCarthy and Smyth, conventional significance testing often fails to ensure that observed differential expression exceeds a biologically meaningful threshold, thereby limiting the translational utility of such analyses in therapeutic development (McCarthy and Smyth, 2009). Similarly, while widely used public repositories like the Gene Expression Omnibus (GEO) provide indispensable access to high-throughput functional genomic datasets and integrated analytical tools such as GEO2R for initial differential expression screening (Barrett et al., 2013), these platforms are typically not designed for the integrative, context-aware prioritization required for drug target identification. Likewise, comprehensive protein-protein interaction resources such as the STRING database, which systematically aggregates and scores interactions from diverse sources including text mining, experimental assays, and computational predictions (Szklarczyk et al., 2023), provide valuable network context but often lack the necessary integration of disease-specific mutational, transcriptional, and pharmacological evidence to robustly prioritize therapeutically actionable targets.

## The GETgene-AI framework: architecture and innovation

In response to these challenges, GETgene-AI is built around an innovative tripartite architecture termed the GE·T strategy. This core methodology synthesizes three complementary and biologically grounded data streams: a Gene (G) list compiled from cancer genomics databases to capture genes with elevated mutational frequencies and functional annotations; an Expression (E) list constructed from disease-specific transcriptomic profiles to identify genes with significant dysregulation in the tumor microenvironment; and a Target (T) list curated from pharmacological and clinical trial resources to incorporate genes with established or potential roles as drug targets. However, the use of clinical trial frequency as an implicit weighting factor—while enhancing druggability considerations—carries the risk of reinforcing historical research biases. As Grudman and colleagues recently demonstrated, researchers have concentrated much of their attention on a limited number of disease-associated proteins, overlooking many important therapeutic targets yet to be explored (Grudman et al., 2025). This research bias, which can be quantitatively assessed through literature analysis, protein interaction data, and diverse sources of clinical, genetic, and molecular information, systematically favors well-studied proteins while leaving understudied but biomedically important targets neglected (Grudman et al., 2025). Recent meta-research has demonstrated that even when high-throughput technologies routinely identify novel genes associated with biological processes and disease, these understudied genes are systematically abandoned in favor of better-studied genes between the completion of -omics experiments and the reporting of results (Richardson et al., 2024). This “leaky pipeline” phenomenon suggests that targets with fewer investigational drugs but novel biological mechanisms may be disadvantaged in early-stage screening through weighting schemes that rely on historical research patterns (Richardson et al., 2024). Thus, the framework must balance its reliance on historical data with safeguards against the systematic abandonment of understudied but potentially important targets—a challenge that resonates with broader concerns in contemporary scientific practice, where claimed research findings may sometimes merely reflect prevailing biases rather than underlying biological truth (Ioannidis, 2005). This integrative design ensures that candidate targets are evaluated through a convergent lens of genetic alteration, transcriptional dysregulation, and pharmacological tractability, thereby enhancing the biological plausibility and clinical relevance of the resulting prioritizations.

## Case study: target prioritization in pancreatic ductal adenocarcinoma

The practical efficacy of GETgene-AI was rigorously demonstrated through a detailed case study in PDAC, a cancer type characterized by complex genetics and limited therapeutic options. The framework successfully identified and ranked several high-confidence targets with strong experimental validation. However, it is important to note that the current GETgene-AI demonstration relies on a single primary PDAC expression cohort for transcriptomic analysis, lacking cross-validation across multi-center, multi-platform datasets. This limitation is particularly significant given recent evidence that platform selection can fundamentally alter biological interpretations in PDAC. A systematic comparison between whole transcriptome and exome capture RNA-seq revealed subtype agreement of only 81%, with exome capture methods failing to achieve statistically significant survival differences between basal-like and classical tumors (log-rank P = 0.061) that were clearly distinguishable using whole transcriptome data (log-rank P < 0.0001) (Lee et al., 2025). These findings demonstrate that technical artifacts arising from platform choice can obscure clinically meaningful prognostic associations. Furthermore, comprehensive clinico-genomic analyses have emphasized that most PDAC genomic studies to date have been constrained by biased cohort compositions and limited clinical information, highlighting the need for large-scale, unbiased datasets that integrate detailed clinical data (Jung et al., 2026).

Notably, PIK3CA emerged as the top-ranked gene—a finding that aligns with established experimental evidence demonstrating that activating mutations in PIK3CA can initiate pancreatic tumorigenesis in murine models and confer sensitivity to dual PI3K/mTOR inhibitors, highlighting its potential as a therapeutic target (Payne et al., 2015). However, from a translational perspective, it is important to acknowledge that while more than 40 compounds targeting the PI3K-AKT-mTOR pathway have been tested in clinical trials, many have not advanced to late-phase randomized studies, and those evaluated in comparative prospective trials have typically shown limited antitumor activity or prohibitive toxicities (Janku et al., 2018). In pancreatic cancer specifically, this disconnect between preclinical promise and clinical reality underscores the challenges of translating genomically prioritized targets into effective therapies, reinforcing the need for predictive biomarkers such as PIK3CA mutations and rational combination strategies to optimize treatment effectiveness (Janku et al., 2018).

Equally compelling was the high ranking of SRC, supported by recent mechanistic studies showing that pharmacological inhibition of Src can reactivate chemotherapy-induced pyroptosis in chemoresistant pancreatic cancer models by modulating the β5-integrin/Src/STAT3/ASAH2 signaling axis, thereby overcoming a key resistance mechanism and restoring therapeutic response (Su et al., 2023). Despite these promising mechanistic insights, clinical experience with SRC inhibitors in solid tumor malignancies has shown little activity in monotherapy trials in unselected patient populations (Puls et al., 2011). Although Src is believed to play an important role in cancer, and several agents targeting Src are in clinical development, combination studies and biomarker-driven clinical trials are still needed to realize their therapeutic potential (Puls et al., 2011). These failed or inconclusive trials serve as important reminders that computational prioritization must be complemented by rigorous translational research addressing resistance mechanisms, pathway compensation, and drug delivery challenges. These examples underscore the framework’s ability to prioritize targets that are not only genomically salient but also mechanistically linked to therapeutic resistance and vulnerability. However, from a translational perspective, it is important to acknowledge that inhibitors targeting the PI3K/AKT/mTOR pathway in PDAC have shown limited single-agent activity in multiple clinical trials, partly due to feedback activation loops and the complex tumor microenvironment (Janku et al., 2018). Similarly, while SRC inhibition shows promise in overcoming chemoresistance, historically, SRC inhibitors have demonstrated disappointing single-agent efficacy in solid tumors (Puls et al., 2011). These failed trials serve as important reminders that computationally prioritized targets must still overcome challenges related to resistance, pathway compensation, and drug delivery in clinical translation.

## Methodological limitations: functional dependency and prospective validation

Beyond these clinical translation challenges, the GETgene-AI framework exhibits two additional methodological limitations that constrain its predictive utility. First, the validation pipeline lacks integration of functional dependency data. As demonstrated by large-scale initiatives such as the Cancer Dependency Map, systematic identification of cancer vulnerabilities requires direct functional interrogation through genome-scale loss-of-function screens (Tsherniak et al., 2017). Analyzing 501 RNAi screens across diverse cancer cell lines, Tsherniak and colleagues developed the DEMETER framework to segregate on-from off-target effects and identified 769 genes that were differentially required in subsets of these cell lines (Tsherniak et al., 2017). Critically, they found that many dependencies could be predicted by molecular features, with expression-based biomarkers being most prominent (Tsherniak et al., 2017). This work establishes that genes essential for cancer cell survival often do not correspond to those identified through genomic or transcriptomic profiling alone, underscoring the necessity of integrating functional dependency data into target prioritization pipelines.

The importance of functional approaches has been further reinforced by more recent CRISPR-Cas9 screens. Behan and colleagues performed genome-scale CRISPR-Cas9 screens in 324 human cancer cell lines across 30 cancer types and developed a data-driven framework to prioritize candidates for cancer therapeutics (Behan et al., 2019). By integrating cell fitness effects with genomic biomarkers and target tractability, they systematically prioritized new targets in defined tissues and genotypes, including the identification of the Werner syndrome ATP-dependent helicase as a synthetic lethal target in microsatellite instability-high tumors (Behan et al., 2019). These findings demonstrate that functional genomic screening can overcome limitations—such as the lack of identification of robust targets—that hamper cancer drug development (Behan et al., 2019). The absence of such functional dependency integration in GETgene-AI means that the framework may prioritize genes that are genomically salient but not functionally essential for tumor maintenance, potentially yielding targets with limited therapeutic impact.

Second, and perhaps more critically, the framework currently lacks prospective experimental validation of its prioritized targets. Functional precision oncology offers a complementary paradigm whereby live tumor cells are directly perturbed with drugs to provide immediately translatable, personalized information to guide therapy (Letai et al., 2022). As Letai and colleagues argue, traditional precision oncology relies on static features of tumors—such as gene expression or genomic mutations—to dictate therapy selection, yet a surprisingly small proportion of individuals derive clinical benefit from this static approach (Letai et al., 2022). Functional precision medicine can provide additional information regarding tumor vulnerabilities that is not captured by static genomic profiling alone (Letai et al., 2022). While retrospective alignment with published literature provides initial credibility—as demonstrated by the PIK3CA and SRC examples—true demonstration of predictive utility requires prospective testing in appropriate preclinical models, including patient-derived organoids and xenografts. Without such validation, it remains uncertain whether GETgene-AI’s rankings genuinely predict therapeutic vulnerability or merely recapitulate existing knowledge encoded in its training data. Yet they also highlight the critical distinction between target identification and therapeutic success—a gap that requires not only computational innovation but also deep biological understanding and clinical insight.

## AI-enhanced literature synthesis: the role and limitations of GPT-4o

A distinctive and forward-looking feature of GETgene-AI is its incorporation of the large language model GPT-4o to automate and enhance the literature review process. This component systematically scores genes based on documented functional significance, research prominence, and therapeutic evidence extracted from a curated corpus of scientific abstracts. This approach resonates with recent methodological evaluations of GPT-4 in systematic review contexts, which suggest that, under optimized conditions—such as the use of carefully designed prompts for full-text screening—large language models can achieve performance levels comparable to human reviewers in tasks such as literature screening and data extraction, though such applications still necessitate careful validation and oversight (Khraisha et al., 2024). By integrating this AI-driven literature assessment, GETgene-AI substantially accelerates a traditionally slow and labor-intensive step while preserving a strong correlation with network-derived biological relevance, thereby adding a robust knowledge-based layer to the prioritization pipeline. However, the incremental methodological value of GPT-4o warrants cautious assessment. Recent comprehensive evaluations of large language models in biomedical applications reveal that while decoder-only architectures like Llama and GPT have become predominant, their effectiveness varies significantly across task types (Cao et al., 2025). Specifically, in biomedical natural language processing tasks, traditional fine-tuned models such as BERT or BART continue to outperform zero-shot or few-shot LLMs in most applications, with GPT-4 excelling primarily in reasoning-related tasks such as medical question answering (Chen et al., 2025). Critically, these evaluations have identified persistent challenges including information inconsistencies, missing content, and hallucinations in LLM outputs, underscoring the necessity of careful validation even when these models are used for literature screening (Chen et al., 2025). Currently, GPT-4o primarily accelerates literature screening and scoring but does not directly contribute to the final ranking score (RP score) calculation. This positions the model as an efficient pre-processing tool rather than a core analytical engine. Future iterations should explore deeper integration of LLM-extracted knowledge—such as complex regulatory logic or mechanistic insights—into quantitative ranking models, moving beyond simple literature evidence counting and addressing the documented limitations of LLM reliability in biomedical contexts (Cao et al., 2025; Chen et al., 2025).

## Network-based expansion and the risk of hub gene inflation

Furthermore, the framework leverages the Biological Entity Expansion and Ranking Engine (BEERE), a dedicated web-based tool designed to help researchers characterize and explore lists of genes or biomedical terms within the context of existing literature and interaction networks (Yue et al., 2019). BEERE performs several critical functions: it assesses the credibility of known associative relationships among entities, ranks entities based on their computed importance within the provided set, and facilitates the generation of novel functional hypotheses through interactive visualization of entity-relationship networks. By utilizing BEERE’s network propagation and ranking algorithms, GETgene-AI effectively embeds candidate genes within their broader interactomic and functional contexts, prioritizing targets based on network centrality and connectivity, and thereby mitigating the noise inherent in high-throughput genomic datasets. However, this network centrality-based ranking carries an inherent risk of “hub gene inflation” (Payne et al., 2015). As Barabási and colleagues note, while network medicine offers a powerful platform for identifying disease modules and pathways, the relationship between a gene’s topological prominence and its disease relevance is not always straightforward (Barabási et al., 2011). Highly connected genes—such as those involved in broad signaling cascades—may naturally dominate network-based rankings due to their centrality, yet this topological feature does not necessarily equate to therapeutic targetability in specific cancer contexts such as PDAC. In fact, disease-associated genes often reside in specific “disease modules” rather than at the network’s most highly connected hubs (Barabási et al., 2011). This topological bias may obscure biologically critical but less-connected genes that function within disease-relevant modules, potentially introducing selection bias into the prioritization pipeline.

## Summary

In conclusion, GETgene-AI represents a methodologically interesting and potentially valuable contribution to the computational landscape of drug discovery. By seamlessly integrating multi-omics data streams, network-based functional expansion through tools like BEERE, and AI-augmented knowledge synthesis via GPT-4o, it establishes a scalable, evidence-based, and interpretable platform for target prioritization. The framework not only addresses the inherent limitations of conventional unidimensional analyses but also provides a flexible and generalizable architecture that can be adapted to diverse cancer types and complex diseases.

However, as the preceding discussion has highlighted, several important limitations warrant consideration. The framework’s reliance on network centrality carries an inherent risk of hub gene inflation; its weighting of clinical trial frequency may reinforce historical research biases; the current lack of functional dependency data integration and prospective experimental validation limits its predictive utility; and the reliance on single-cohort, single-platform transcriptomic data raises concerns about generalizability. Moreover, while GPT-4o enhances literature review efficiency, its role remains that of a preprocessing tool rather than a core analytical engine, and the documented challenges of LLM reliability—including information inconsistencies and hallucinations—necessitate continued caution.

These limitations do not diminish the framework’s value but rather situate it within the broader landscape of computational target discovery, where no single approach can capture the full complexity of therapeutic vulnerability. The true test of GETgene-AI’s utility will lie not in retrospective alignment with known biology but in its prospective ability to predict novel, therapeutically actionable targets that translate into clinical benefit.

As artificial intelligence continues to mature and permeate biomedical research, integrated frameworks like GETgene-AI may play an increasingly important role in bridging the gap between large-scale genomic data and actionable therapeutic hypotheses. Realizing this potential will require ongoing methodological refinement, integration of functional genomic data, rigorous prospective validation, and critical awareness of the biases and limitations inherent in any computational approach. Ultimately, frameworks like GETgene-AI contribute to the accelerating evolution of precision oncology, but they must be viewed as complements to—not substitutes for—deep biological understanding and clinical insight.
